# Biodeterioration of plasma pretreated LDPE sheets by *Pleurotus ostreatus*

**DOI:** 10.1371/journal.pone.0203786

**Published:** 2018-09-13

**Authors:** Luis D. Gómez-Méndez, Diana A. Moreno-Bayona, Raúl A. Poutou-Piñales, Juan C. Salcedo-Reyes, Aura M. Pedroza-Rodríguez, Andrés Vargas, Johan M. Bogoya

**Affiliations:** 1 Laboratorio de Microbiología Ambiental y Suelos, Grupo de Biotecnología Ambiental e Industrial (GBAI), Departamento de Microbiología, Facultad de Ciencias, Pontificia Universidad Javeriana, Bogotá, D.C., Colombia; 2 Laboratorio de Biotecnología Molecular, Grupo de Biotecnología Ambiental e Industrial (GBAI), Departamento de Microbiología, Facultad de Ciencias, Pontificia Universidad Javeriana, Bogotá, D.C., Colombia; 3 Laboratorio de Películas delgadas, Grupo de Películas Delgadas y Nanofotónica, Departamento de Física, Facultad de Ciencias, Pontificia Universidad Javeriana, Bogotá, D.C., Colombia; 4 Grupo de Física-Matemática, Departamento de Matemáticas, Facultad de Ciencias, Pontificia Universidad Javeriana, Bogotá, D.C., Colombia; VIT University, INDIA

## Abstract

Low-density polyethylene (LDPE) waste generates an environmental impact. To achieve the most suitable option for their degradation, it is necessary to implement chemical, physical and biological treatments, as well as combining procedures. Best treatment was prognosticated by Plackett-Burman Experimental Design (PB), evaluating five factors with two levels (0.25 mM or 1.0 gL^-1^ glucose, 1.0 or 2.0 mM CuSO_4_, 0.1 or 0.2 mM ABTS [2, 20-azino-bis(3-ethylbenzothiazoline-6-sulphonic acid)], pH 4.5 ± 0.2 or 7.0 ± 0.2 and 30 or 90 day incubation), which was reproduced for 150 days. Therefore, PB identified a sequential treatment (plasma followed by fungus) for partial LDPE biodeterioration. Sheets were pretreated with glow discharge plasma (O_2_, 3.0 x 10^−2^ mbar, 600 V, 6 min.), followed by *Pleurotus ostreatus* biodeterioration. Fungus growth, colonization percentage, and pigment generation followed. Laccase (Lac), manganese peroxidase (MnP) and lignin peroxidase (LiP) activities were appraised. Additionally, contact angle (CA), functional group presence and changes and carbonyl and vinyl indices (Fourier transformed infrared spectroscopy) were evaluated. LDPE surface changes were assessed by Young’s modulus, yield strength, scanning electronic microscopy (SEM), Fourier transformed infrared spectroscopy (FTIR) and atomic force microscopy (AFM). Plasma discharge increased hydrophilicity, decreasing CA by 76.57% and increasing surface roughness by 99.81%. *P*. *ostreatus* colonization was 88.72% in 150 days in comparison with untreated LDPE (45.55%). After this treatment carbonyl groups (C = O), C = C insaturations, high hydrophilicity CA (16 ± 4) °, and low surface roughness (7 ± 2) nm were observed. However, the highest Lac and LiP activities were detected after 30 days (Lac: 2.817 U Lac g^-1^ and LiP: 70.755 U LiP g^-1^). In addition, highest MnP activity was observed at day 120 (1.097 U MnP g^-1^) only for *P*. *ostreatus* treated LDPE. Plasma favored *P*. *ostreatus* adsorption, adherence, growth and colonization (88.72%), as well as partial LDPE biodeterioration, supported by increased hydrophilicity and presence of specific functional chemical groups. The approximate 27% changes in LDPE physical properties support its biodeterioration.

## Introduction

Low-density polyethylene (LDPE) is one of the most used polymers for synthetic material production, such as plastic bags [1], bottles, pipes and various laboratory materials [2], among others. Due to increased worldwide LDPE residues, approximately 34 million tons, decreased landfill capacity, and poor plastic degradation research has focused on LDPE treatment alternatives to reduce its presence in soils and water bodies [3]. At present biodegradable plastic bags are available, made-up of natural polymers easily degraded by microorganisms. Other alternatives include oxo-biodegradable polymers derived from petroleum that contain special additives, such as totally degradable plastic additive TDPA^™^. Additionally, to facilitate LDPE degradation a master batch containing polymers and dispersed additives on a carrier resin, such as d2w^™^ has been implemented [4]. Despite these efforts, use of biodegradable bags is not popular in many countries, since its availability is restricted to certain sectors of society [5].

LDPE polymer it is not easily to degrade, due to its high molecular weight, 3D structure, hydrophobic nature and absence of polar functional groups [6]. Never the less, some microorganims can play an important role in decomposing this type of material [1].

LDPE physicochemical treatments favor deterioration [7]. UV irradiation (photo-oxidation), thermo-oxidation and polyethylene chemical oxidation, as well as previous exposure to biotic conditions increase biodeterioration [8], since material chemical modification takes place, making it accessible to some microorganisms. One of these physicochemical treatments is plasma discharge. This treatment can alter hydrophobicity and surface roughness of many polymers, modifying surface adherence properties [9]. This is ensued by chemical changes generating polar groups, such as carboxyl (-COOH) and hydroxyl (-OH), in addition to crosslink formation of ramified chains of the polymer [10,11]. Moreover, oxidized structures of low molecular weight on the plastic surface can be generated [12,13]. These modifications make LDPE more accesible to microorganism attack, which biodeteriorate these materials by extracellular enzyme production. Chemical changes convert LDPE into small soluble compounds easy to absorb [14], such as carboxylic acids that once inside the cell can be metabolized through β-oxidation and Krebs cycle [15]. *Pleurotus ostreatus* white rot fungi is among these microorganisms. Rodrigues da Luz et al., (2013, 2015) has reported *Pleurotus ostreatus* as degrader of oxo-biodegradablede LDPE and green LDPE (polymer synthesized from sugar cane), [16,17]. *Pleurotus ostreatus* hyphae adsorb and adhere to LDPE through exopolysaccharides and a thin network of fungal mycelia [6], facilitating material colonization. In addition, *P*. *ostreatus* makes part of white rot fungi producing enzymes, such as manganese peroxidase (MnP, 1.11.1.13), [18] and laccases (Lac, EC. 1.10.3.2), [19]. This latter one degrades a broad range of aromatic organic compounds [19]. Its activity can be extended to non-aromatic compounds when used with mediators, such as ABTS (2,2’-azinobis(3-ethylbenzothiazoline-6-sulfonate)) and HBT (1-hydroxy-benzotriazole) [19]. As a case in point, for polyethylene (PE) and nylon 66 degradation, Fujisawa et al., (2001) employed a laccase mediated system [20].

Initial high molecular weight (MW) polymer deterioration, such as LDPE requires enzyme hydrolysis, generating a functional group favoring hydrophilicity. Subsequently, LDPE chains are degraded resulting in low MW polymers of weak properties, making them more accessible to microbial attack [21]. Last, under aerobic conditions final degradation products are CO_2_ and H_2_O [22]. Understanding of biological mechanisms derived from PE degradation is poor, since most reports describe observations on material physical and chemical changes [23].

The present study investigated white rot fungi *Pleurotus ostreatus* as a LDPE traditional sheet biodeteriorator. LDPE was previously treated with plasma oxygen (O_2_) luminescent discharge in a wet chamber in Radha semisolid modified media. Changes associated with fungal growth and colonization were analyzed and lignolytic enzyme production was assayed. Physical, chemical and mechanical properties of LDPE were evaluated.

## Results

### Pretreatment of LDPE with oxygen glow discharge plasma

**Fig 1:**
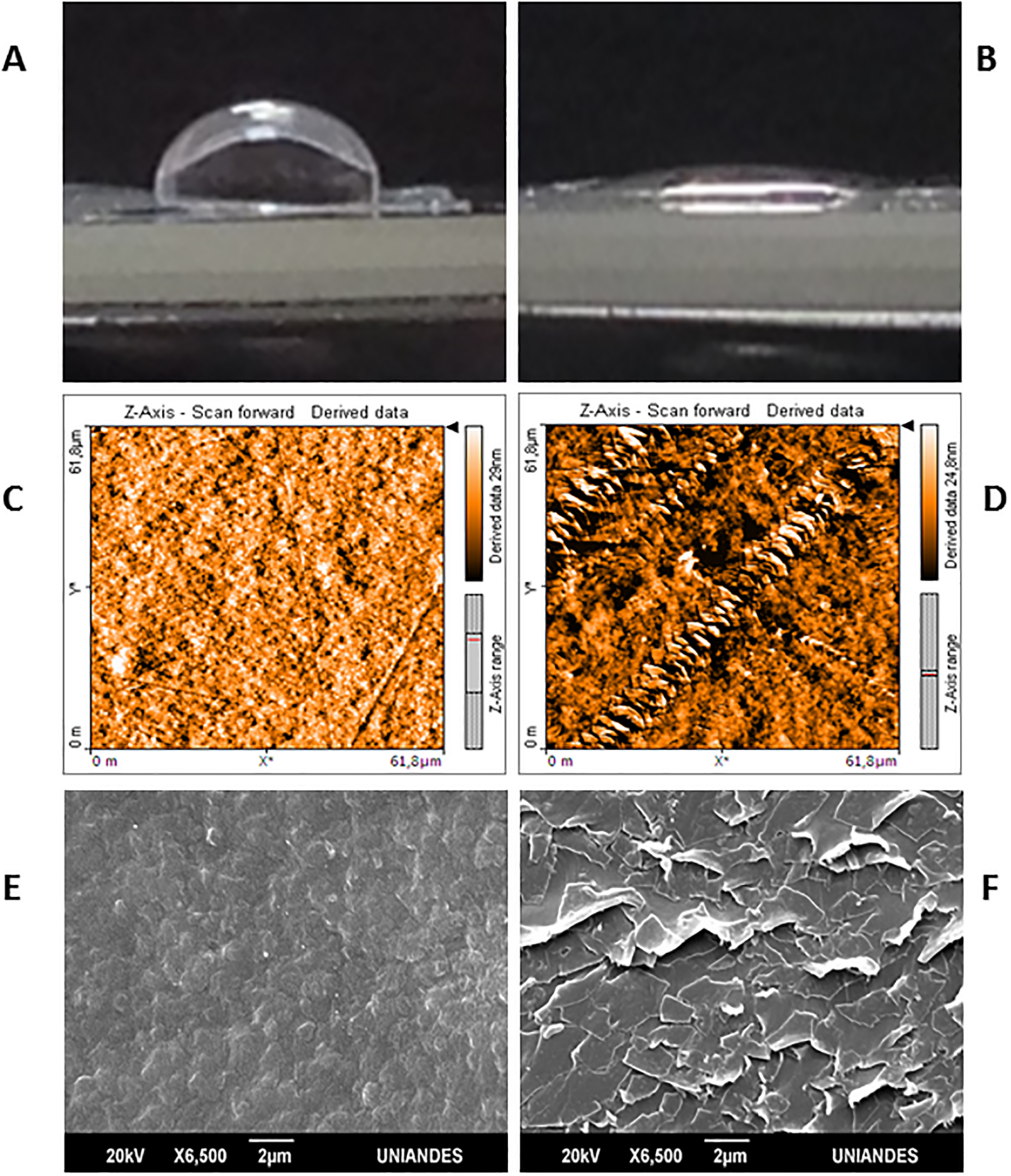
Contact angle photograph. Pristine LPDE (A). After oxygen plasma, treatment a decrease in contact angle was observed (B). Pristine LDPE. A homogenous surface was appreciated, with discrete roughness (**C**). LDPE pre-treatment with oxygen plasma discharge. Areas with tears were observed, due to ablation process (**D**). Pristine LPDE SEM photograph (E). After treatment cracks, tears and fissures were observed through SEM (F).

This work employed plasma as pre-treatment for LDPE *P*. *ostreatus* biodeterioration. After pre-treatment a 75% decrease in material hydrophobicity was observed, denoting an attained 25% gain in hydrophilicity (Fig 1A and 1B). In addition, a change in surface roughness was observed (starting with 5 ± 1 nm and ending with 10 ± 3 nm), representing a 5 ± 4 nm increase in roughness, due to LDPE surface ablation (Fig 1E and 1D). Table 1 describes pristine LDPE analyses after plasma discharge pre-treatment.

**Table 1:** Physical and chemical changes associated with LPDE sheets before and after plasma discharge pre-treatment (O_2_, 3.0 x 10^−2^ mbar pressure, and 600 V voltage).

Response variable	Pristine LDPE	Post-plasma LDPE	References
Weight (mg)	4.3 ± 0.5	4.3 ± 0.1	Present work
Static contact angle (°)	87 ± 1	21 ± 3	Present work
Roughness (nm)	5 ± 1	10 ± 3	Present work
Young´s modulus (Mpa)	34 ± 6	41 ± 6	Present work
Yield strength (Mpa)	10 ± 2	11 ± 2	Present work
Vibrational state (FTIR)	729 cm^-1^ and 719 cm^-1^ CH_2_ deformation balance	Signal decrease	[25,26]
	1471 cm^-1^ and 1462 cm^-1^ CH_2_ flexion band	Signal decrease	[25,27]
	2921 cm^-1^ and 2843 cm^-1^ asymmetric and symmetric CH_2_ stretching respectively	Signal decrease	[25,26]
	ND	Signal decrease at 906 cm^-1^: vinyl groups	[6,27]
	ND	Signal appearance at 1175 cm^-1^: C-OH bond stretching vibration	[28]

ND: not detected

### Plackett-Burman experimental design (PB) for condition selection favoring plasma pretreated LDPE sheet biodeterioration by *P*. *ostreatus*

After 12 treatment of PB, SCA and surface roughness were the only factors statistically significant in comparison with untreated controls as determined by ANOVA analysis (p = 0.049 and p = 0.0011, respectively). Therefore, the effect of PB factors on these two variables was evaluated. Predicted and observed values had a high correlation (R^2^ = 0.8500 and 0.8913, respectively, an adequate precision values were greater than 4.0 (5.98 and 12.68), confirming results were not due to experimental error. Table 2 presents model significance, values, factors (Prob > f), effects and contribution percentages for each factor.

**Table 2:** LDPE contact angle and surface roughness Plackett Burman response variables (ANOVA).

Static Contact Angle (SCA)					Surface Roughness				
Factor	F value	Prob> f	Stand. Effect	Contribution %	Factor	F value	Prob>f	Stand. Effect	Contribution %
		*p* value					*p* value		
Model	5.6	**0.049**	35.08		Model	13.12	**0.011**	4.33	
A-Glucose	5.8x10-3	0.941	0.1	0.05	A-Glucose	0.2	0.6679	0.04	0.2
**B-CuSO4**	2.74	**0.014**	-2.21	22.45	B-CuSO_4_	2.84	0.1539	0.13	2.61
C-ABTS	3.0x10-6	0.999	-2.3x10-3	2.1x10-5	**C-ABTS**	19.91	**0.0021**	-0.38	20.94
**D-pH**	1.5	**0.041**	-0.17	0.12	**D-pH**	32.09	**0.0005**	-0.48	33.76
**E-Time**	3.3	**0.037**	-0.23	0.24	**E-Time**	10.91	**0.0108**	-0.28	11.47
Curvature F-value	1.41	0.269			Curvature F-value	21.46	**0.0017**		
Lack of fit	8.10	0.114			Lack of fit	1.24	0.5101		

**In bold**: significant values for the model and factors that resulted significant in the model

Factors having an effect on SCA decrease were CuSO_4_, pH and exposure time (p = 0.0136, p = 0.041 and p = 0.037, respectively). The one with the highest contribution percentage was CuSO_4_ (22.45%), followed by exposure time (0.24%) and pH (0.12%). Copper sulfate PBED value was negative, indicating a lower value could be assayed (1.5 mM). Glucose and ABTS were not significant (p > 0.05).

For surface roughness factors having an effect were ABTS concentration, pH and exposure time (p = 0.0021, p = 0.0005 and p = 0.0108, respectively), with contribution percentages of 20.9%, 33.87% and 11.4%, respectively. Additionally, standardized effects and their respective signs were -0.48, -0.38 and -0.28, specifying the three factors could be lowered to favor increased LDPE surface roughness in the sequential plasma and *P*. *ostreatus* process.

**Fig 2:**
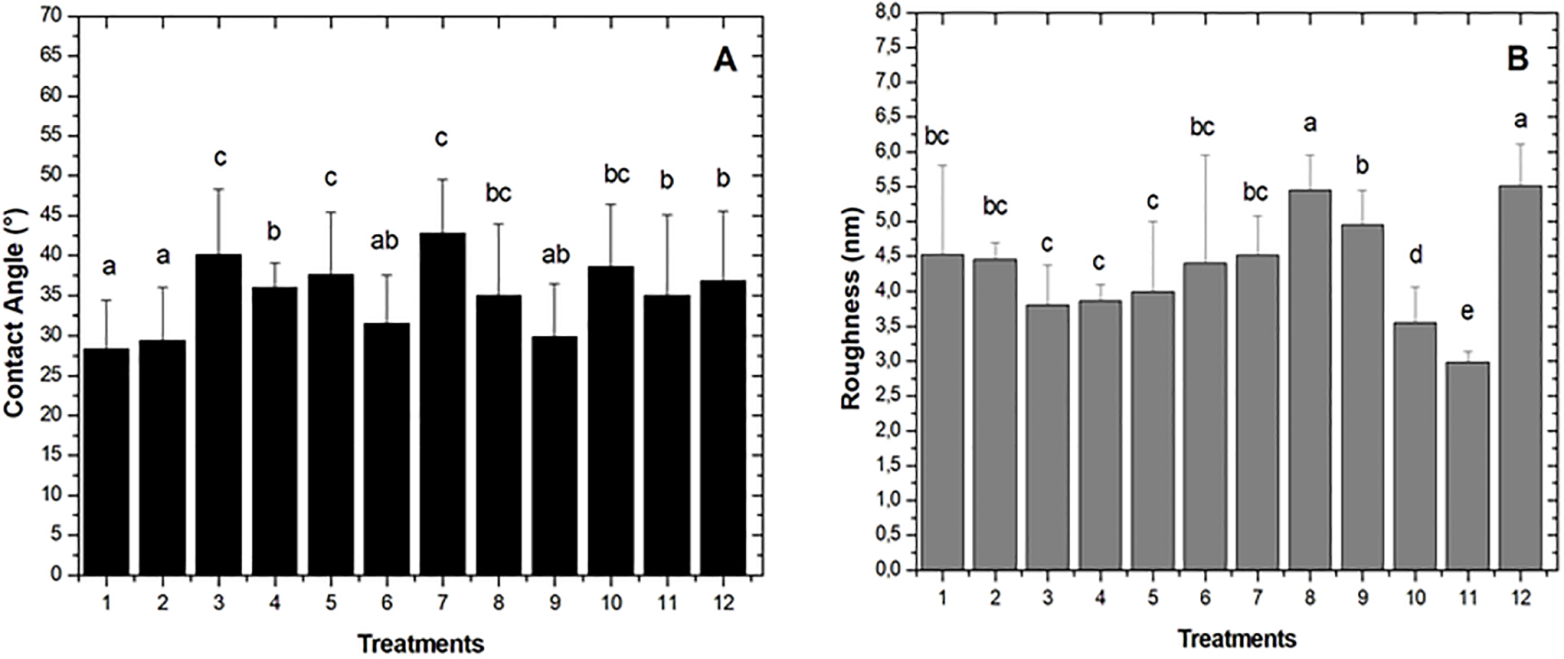
Comparison of means of contact angle and surface roughness PBED treatments. (**A**) For SCA the most significant treatments were T1 (28 ± 6)° and T2 (29 ± 7)°. (**B**) For surface roughness both T8 and T12 were the most significant with 5 ± 1 nm.

Based on ANOVA analysis (Table 2), Tukey *post hoc* test (Fig 2), and considering SCA principal response value, it was decided to elect T2 (modified semisolid Radha agar: 0.625 gL^-1^glucose, 1.5 mM CuSO_4_, 0.1 mM ABTS and pH 5.75 ± 0.2. In addition, 2 gL^-1^ KH_2_PO_4_, 0.05 gL^-1^ NH_4_Cl, 0.5 gL^-1^ MgSO_4_ H_2_O, 0.1 gL^-1^ CaCl_2_ 2H_2_O, 10 mL L^-1^ and trace element solution (0.5 gL^-1^ MnSO_4_, 0.1 gL^-1^ FeSO_4_ 7H_2_O, 0.1 gL^-1^ ZnSO_4_ 7H_2_O), 7 gL^-1^ agar-agar), for the 150-day biodeterioration curve.

Fig 2 illustrates means among treatments (Tukey *post hoc* test) for SCA and surface roughness. Treatments T1 and T2 obtained the lowest values SCA (28 ± 6) ° and (29 ± 7) °, respectively, while T8 and T12 treatments resulted in higher surface roughens values (5 ± 1 nm).

During PB assay in addition to quantitative variables, green halos around fungal biomass were observed during the first 48 h of incubation followed by diffusible brown pigment around seeded fungal biomass. Moreover, white *P*. *ostreatus* mycelium adhered to the Petri dish, which had a compact appearance with time (S1 Fig).

### 150-days *P*. *ostreatus* biodeterioration assays curves. Variables and analytical techniques associated with LDPE sheet changes (Weight loss, Young´s modulus Yield strength and SCA)

Table 3 presents viscoelastic properties (Young´s modulus and yield strength), roughness and weight result variables, as well as SCA measurements after performing Plackett Burman T2 treatment. Biodeterioration curve at 150 days in Radha semisolid modified media (PBBT_150_) and PT3 and C2 controls.

**Table 3:** LDPE variable response after 150 days *P*. *ostreatus* treatment.

Experimental assay	LDPE condition	Young´s Modulus (Mpa)	Yield Strength (MPa)	Roughness (nm)	Weight (mg)	SCA (°)
Plackett-Burman	T2: LDPE + plasma + *P*. *ostreatus*	59 ± 9	8 ± 1	4 ± 1	4.7 ± 0.5	29 ± 7
Biodeterioration curve	PBBT_150_: LDPE + plasma + *P*. *ostreatus*	48 ± 4	8 ± 1	7 ± 2	4.5 ± 0.4	17 ± 4
	PT3: LDPE + plasma	54 ± 5	8 ± 1	10 ± 3	4.4 ± 0.5	21 ± 3
	C2:LDPE prístino	34 ± 6	10 ± 2	5 ± 1	4.4 ± 1.0	89 ± 1

### Fourier transformed infrared spectroscopy (FTIR) analysis

**Fig 3:**
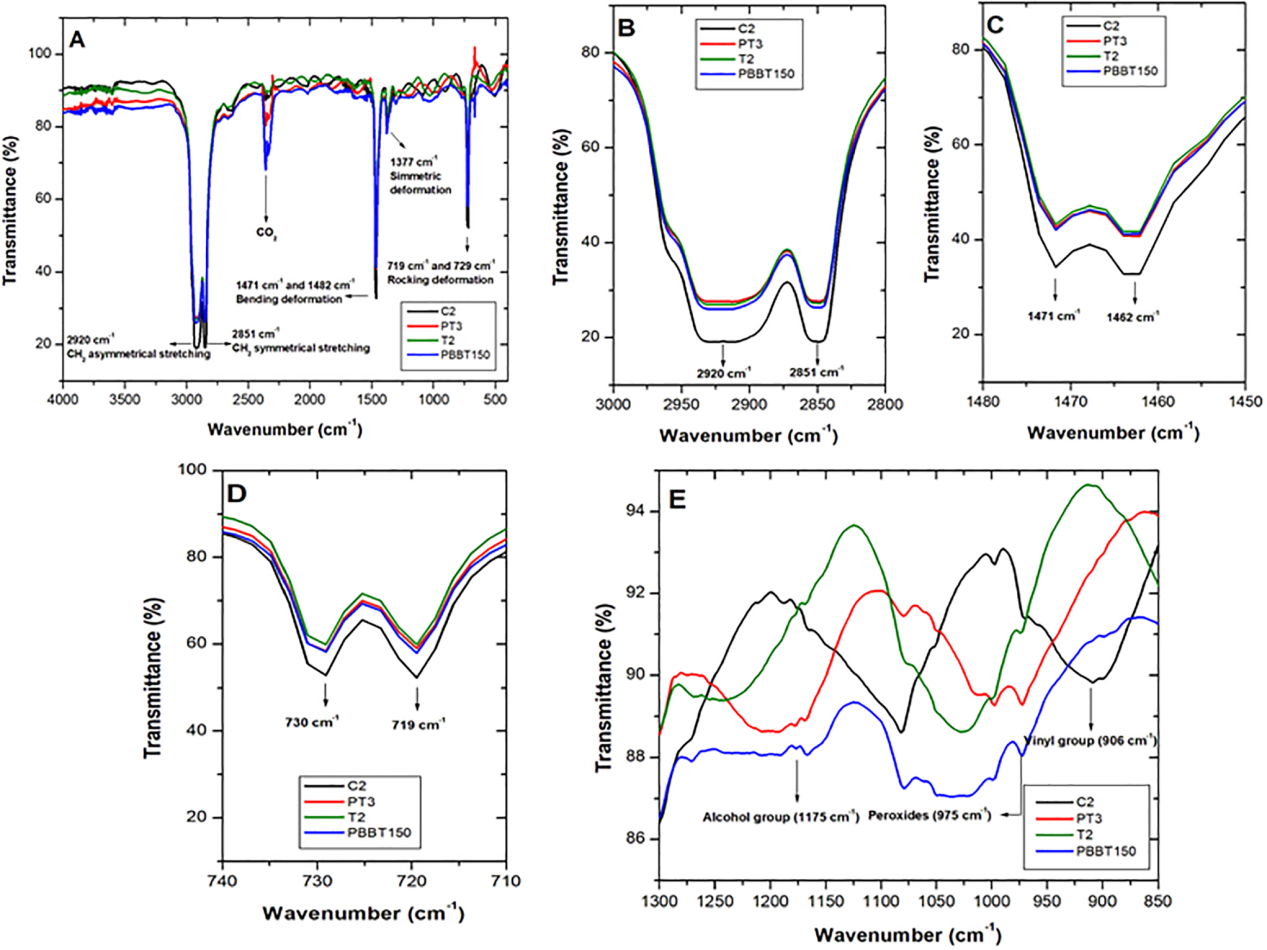
Fourier transformed infrared spectroscopy (FITR) for pristine LDPE (control C2, black), LDPE + plasma (PT3, red), LDPE PB T2 (T2, green) and LDPE + plasma+ *P*. *ostreatus* at 150 days (PBBT_150_, blue). (**A**) Complete spectrum that show LDPE characteristic signals. (**B, C and D**) Detailed LDPE signal characteristics before and after treatments. (**E**) Signal changes at 909 cm^-1^ corresponding to vinyl groups, 975 cm^-1^ (peroxides groups) and 1175 cm^-1^ (alcohol groups).

Fig 3 shows FTIR for pristine LDPE (C2, black), LDPE + plasma (PT3, red); LDPE-T2 at 90 days PB treatment (T2, green) and LDPE after biodeterioration curve at 150 days (PBBT_150_, blue). Complete spectra illustrate IR bands characteristic of LDPE (Fig 3A) Carbon-Hydrogen (CH) group main chain stretching vibrations at 2920–2846 cm^-1^ and of methylene (CH_2_) bending and rocking vibrations at 1471–1462 cm^-1^ and 731–719 cm^-1^, respectively [24]. When comparing pristine (C2) LDPE characteristic signals with PT3, T2 and PBBT_150_ treatments, changes in their intensities were observed (Fig 3B, 3C and 3D). A decreased signal for treatments at 906 cm^-1^ was observed, corresponding to vinyl groups (-CH = CH2), (Fig 3E), as well as the appearance of a signal at 1175 cm^-1^, corresponding to alcohol groups.

### Carbonyl (Ico) and vinyl indices (Iv)

*ICo* is a measurement of sample C-O and C = O bond proportion. As observed in Table 4 and S3 Fig, pristine LDPE presented an initial I*co* of 1.19086 ± 0.04609, suggesting presence of carbonyl groups from the beginning of the study. After plasma treatment a slight decrease was observed for PBBT_150_ (1.18326 ± 0.04524). It continued decreasing during the biodeterioration curve up to day 60 (1.13596 ± 0.03817). From day 90 up to 150, it remained near 1.15, ending at 1.15017 ± 0.04456. For BT2 a decrease in these groups was recognized up to day 30 (1.11975 ± 0.06019) and ranged between 1.14636 ± 0.04323 and 1.15625 ± 0.02282 for day 120 and 150, respectively.

**Table 4:** LDPE I*co* and I*v* indices behavior during 150-day biodeterioration curve.

**I*co***							
	**Pristine**	**Plasma**	**Day 30**	**Day 60**	**Day 90**	**Day 120**	**Day 150**
**PBBT**_**150**_	1.19086 ± 0.04609	1.18326 ± 0.04524	1.16293 ± 0.04825	1.13596 ± 0.03817	1.15880 ± 0.05701	1.15468 ± 0.03864	1.15017 ± 0.04456
**BT2**	1.19086 ± 0.04609	N.A	1.11975 ± 0.06019	1.15872 ± 0.02683	1.15514 ± 0.02277	1.14636 ± 0.04323	1.15625 ± 0.02282
**I*v***							
	**Pristine**	**Plasma**	**Day 30**	**Day 60**	**Day 90**	**Day 120**	**Day 150**
**PBBT**_**150**_	1.01801 ± 0.02836	1.03815 ± 0.01154	1.00326 ± 0.03735	1.00647 ± 0.02825	1.02418 ± 0.01193	0.99756 ± 0.01619	1.01909 ± 0.01716
**BT2**	1.01801 ± 0.02836	N.A	1.01222 ± 0.04078	1.00424 ± 0.01943	0.99758 ± 0.02840	1.00718 ± 0.02476	1.00077 ± 0.02329

PBBT_150_: physical/biological treatment. BT2: biological treatment. NA: Does not apply.

For pristine LDPE I*v* index was 1.01801 ± 0.02836. After submitting plastic sheets to plasma treatment (PBBT_150_), this index had a slight increase (1.03815 ± 0.01154); augmenting up to 1.02418 ± 0.01193 at day 90 of the biodeterioration curve. A slight decrease was recognized at day 120 (0.99756 ± 0.01619), ending with a 1.01909 ± 0.01716 value at day 150. For treatment BT2 a minimum value at day 90 was obtained (0.99758 ± 0.02840), and a maximum at day 30 (1.01222 ± 0.04078), ending at day 150 with a 1.00077 ± 0.02329 value.

### Computational image processing for *P*. *ostreatus* growth after 150-days biodeterioration assays curves

**Fig 4:**
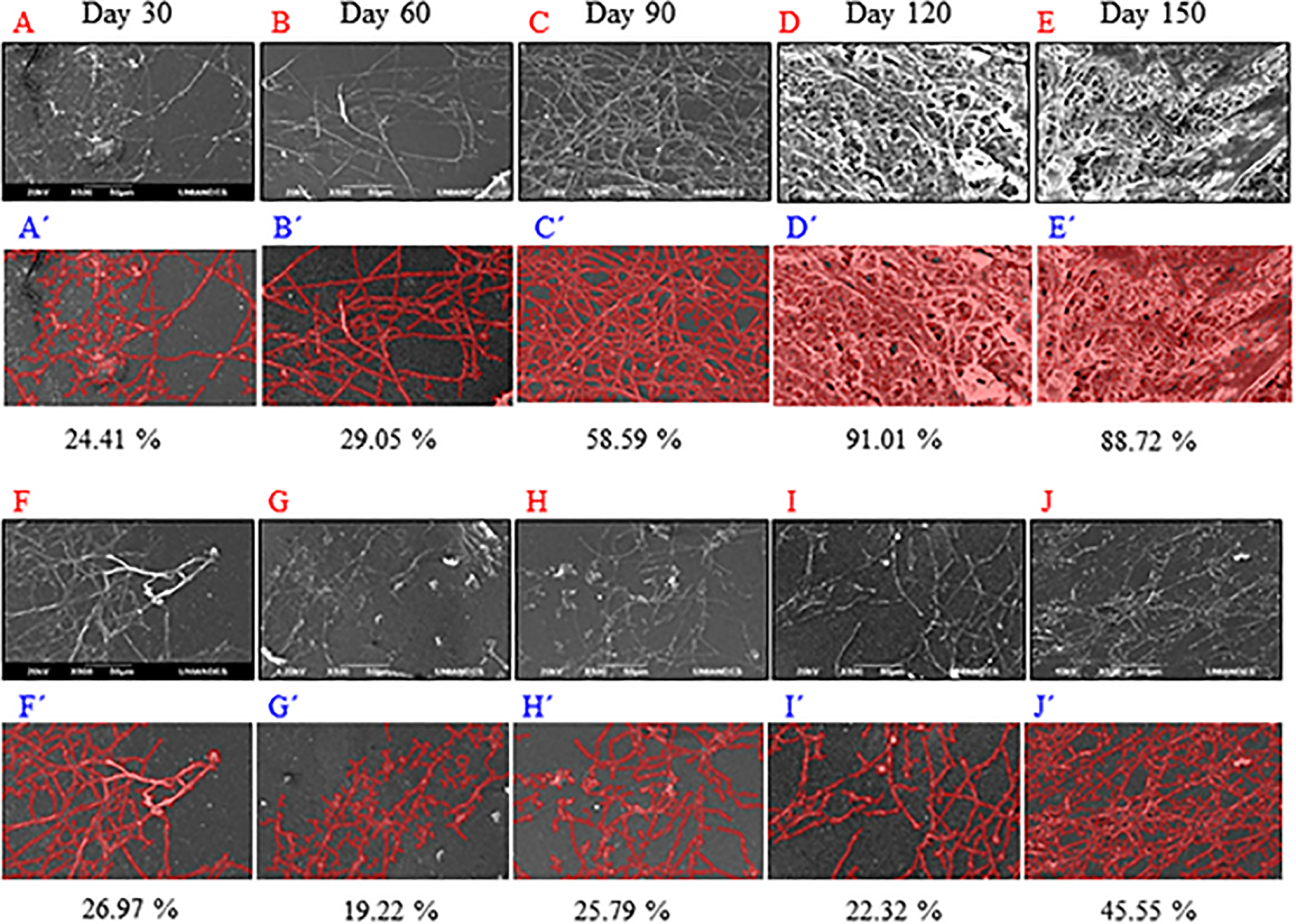
Scanning electron microscopy images of LPDE under physical/biological (PBBT_150_), (A-E) and biological treatment (BT2), (F-J). Image analyses for the same treatments (A’-E’) and (F’-J’). Observations were carried-out every 30 days. Sequential treatment physical/biological favored *P*. *ostreatus* colonization, surpassing 88% at 150 days of incubation. Biological treatment presented a lower adherence and colonization, without surpassing 50% after 5 months of treatment.

Scanning electron microscopy images depict *P*. *ostreatus* growth and colonization on LDPE sheets exposed to physical/biological (PBBT_150_) or physical treatment (BT2), (Fig 4). A greater colonization was observed for PBBT_150_ (88.72%) compared with BT2 (45.55%) at day 150.

### Response variables associated with *P*. *ostreatus* metabolism

Table 5 presents response variables associated with mycelial growth (MG), semi-quantitative Lac activity (first and second ABTS oxidation) melanin production (M), for PBBT_150_ and BT2 treatments and C3 control. Mycelia growth on plastic sheets and semisolid agar was lower in PBBT_150_ and BT2 (27 mm at 150 days) in comparison with control without plastic sheets (31 mm). For 150-day biodeterioration curve biomass production among PBBT_150_, BT2 and C3 presented statistically significant differences [F = 33.295, p = 0.001, (p < 0.05)], (ANOVA). Tukey *post hoc* test revealed significant differences between C3 and PBBT_150_ p = 0.036), between C3 and BT2 (p = 0.001) and between PBBT_150_ and BT2 (p = 0.002).

**Table 5:** Semi-quantitative measurements related to *P*. *ostreatus* metabolic activity in contact with LDPE sheets after 150 days in semi-solid modified Radha media.

	Treatment PBBT_150_				Treatment BT2				Treatment C3			
Time (d)	MG (mm)	A*	A**	P	MG (mm)	A*	A**	P	MG (mm)	A*	A**	P
0	0	5 ± 1	3 ± 0	0	0	4 ± 0	0	0	0	3 ± 1	2 ± 0	0
7	12 ± 4	0	0	1 ± 1	14 ± 3	0	1 ± 1	3 ± 2	25 ± 3	2 ± 1	0	4 ± 2
30	27 ± 2	0	0	2 ± 0	27 ± 2	0	0	8 ± 3	31 ± 3	0	0	5 ± 2
60	27 ± 2	0	0	4 ± 1	27 ± 2	0	0	7 ± 1	31 ± 3	0	0	6 ± 1
90	27 ± 2	0	0	7 ± 1	27 ± 2	0	0	8 ± 3	31 ± 3	0	0	6 ± 1
120	27 ± 2	0	0	7 ± 1	27 ± 2	0	0	7 ± 1	31 ± 3	0	0	6 ± 1
150	27 ± 2	0	0	9 ± 1	27 ± 2	0	0	6 ± 2	31 ± 3	0	0	5 ± 1

MG: mycelial growth; A*: ABTS 1^st^ oxidation (green zone); A**: ABTS 2^nd^ oxidation (purple zone); P: pigments. All measurements are in mm.

Presence of redox mediator (ABTS) allowed visualizing two ABTS oxidation cycles as a consequence of Lac activity. The first oxidation cycle was evidenced during set-up up to day seven of incubation, by green halo formation around fungal biomass. Oxidation halos were 5 ± 1 mm and 4 ± 0 mm, for PBBT_150_ and BT2, respectively. The second oxidation cycle was characterized by purple zones, appeared at the same time, but with smaller oxidation halos (3 ± 0 mm and 1 ± 1 mm), for PBBT_150_ and BT2, respectively. No oxidation halos were observed from the seventh day on, which could indicate ABST was used as a redox mediator, oxidizing LDPE during the first phases of the biodeterioration process.

Pigment production as melanins increased as a function of time. PBBT_150_ and BT2 had the greatest melanin halos: 9 ± 1 mm and 6 ± 2 mm at 150 days, respectively. For C3 these pigments were also observed, but in lesser proportion (melanin halos between 4 and 6 mm) (S2 Fig). Statistical analysis demonstrated significant differences between these conditions [F = 11.023 p = 0.004 (p < 0.05)]. On the other hand, Tukey *post hoc* test did not evidence any significant difference between C3 and PBBT_150_ (p = 0.541). However, significant differences were observed between PBBT_150_ and BT2 (p = 0.004). These results suggest LDPE presence in treatments generated an oxidative stress for *P*. *ostreatus* with greater growth in C3, greater Lac semi-quantitative activity for PBBT_150_ and greater pigment production for BT2.

**Fig 5:**
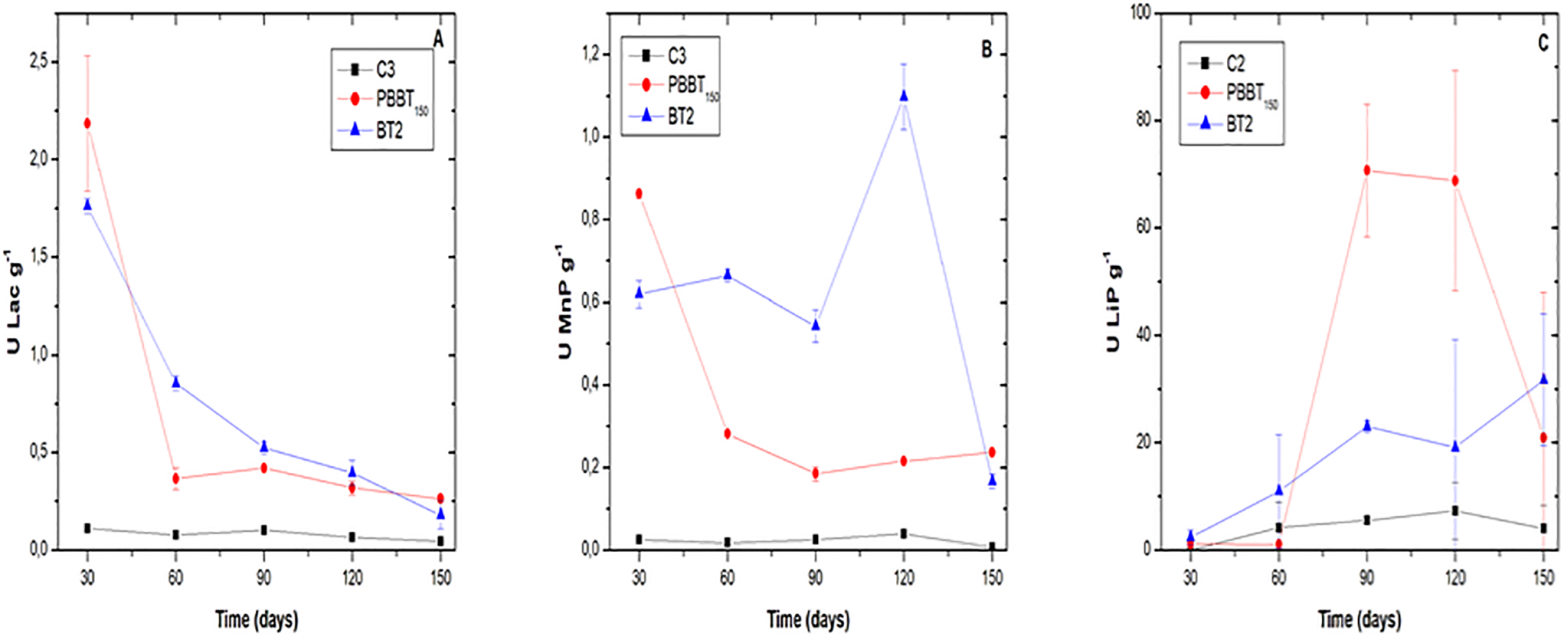
*P. ostreatus* Lac (A), MnP (B) and LiP (C) enzyme activities quantified during 150 days for PBBT_150_ (LDPE + plasma + *P*. *ostreatus*), BT2 (LDPE + *P*. *ostreatus*) and C3 (*P*. *ostreatus* growing in Radha media).

Fig 5 presents lignolytic enzyme activities during five months in semisolid Radha modified media in the presence of LDPE sheets. Enzymes capable of lignin degradation have also been reported to participate in PE biodeterioration, including Lac, MnP and LiP [29,30].

A greater Lac enzyme production was observed during the first 30 days (Fig 5A), with an enzyme activity of 2.8167 U Lac g^-1^ for PBBT_150_ and 1.760 U Lac g^-1^ for BT2 in comparison with a 0.110 U Lac g^-1^ activity for C3. It then decreased during the following days until reaching 150 days with enzymes activities of 0.263 U Lac g^-1^, 0.181 U Lac g^-1^ and 0.045 U Lac g^-1^, for PBBT_150_, BT2 and C3, respectively. Statistical analysis revealed significant differences (p < 0.05) among PBBT_150_, BT2, and C3 for day 30 (p = 0.001), for day 60 (p = 0.001) and day 90 (p = 0.001). For days 120 and 150 significant differences were observed between both treatments and the control (p < 0.05).

MnP enzyme activity (Fig 5B) presented significant differences among PBBT_150_, BT2 and C3 during the five sample collection intervals (p < 0.05). BT2 presented higher enzyme activities compared with PBBT_150_ for days 30 and 150, with activities of 0.862 U MnP g^-1^ and 0.236 U MnP g^-1^, respectively, whereas for PBBT_150_ they were 0.619 U MnP g^-1^ and 0.167 U MnP g^-1^ (p = 0.001), respectively. In contrast, BT2 MnP g^-1^ activity predominated for days 60, 90 and 120, with activities of 0.665 U MnP g^-1^, 0.543 U MnP g^-1^ and 1.097 U MnP g^-1^, respectively, in comparison with 0.282 U MnP g^-1^, 0.186 U MnP g^-1^ and 0.216 U MnP g^-1^, for PBBT_150_, respectively (p = 0.007).

LiP production (Fig 5C) was the highest among all enzyme activities evaluated. Enzyme activity for PBBT_150_ at 90 and 120 days were 70.755 U LiP g^-1^ and 68.820 U LiP g^-1^, respectively. In contrast for the same days activities were 23.088 U LiP g^-1^ and 19.150 U LiP g^-1^ for BT2, thus presenting significant differences between treatments PBBT_150_ and BT2 at 90 (p = 0.001) and 120 days (p = 0.026).

## Discussion

### Pretreatment of LDPE with oxygen glow discharge plasma

Polymers suffer physical and chemical changes when submitted to glow discharge oxygen plasma treatment, modifying their hydrophobicity, roughness and weight (Table 1). These modifications are based on polar group formation, such as carbonyl (-C = O) and hydroxyl (-OH), [23,29,31], in the polymer’s crosslinking and ramification by the presence of microalkyl radicals [11,32], in addition to oxidized molecule formation of low molecular weight [12,13].

After plasma discharge a non-significant weight variation (0.02 mg, p = 0.5068) in LDPE sheets was observed, thus this response variable was not utilized for posterior analyses. Additionally, viscoelastic properties (Young´s modulus and yield strength) augmented 7.04 Mpa and 1.14 Mpa, respectively (Table 1). Oxygen plasma discharge treatment chemically added on the main carbon chain other atoms or polar groups, hindering LDPE rotation, stretching, untanggling or deformation when a strain was applied, resulting in increased rigidity, in addition to providing stronger Van de Waals bonds between chains [33].

Initially pristine LDPE presented a homogenous surface, (Fig 1E) with a SCA of (87 ± 1)° (Fig 1A) and a mean roughness of (5 ± 1) nm (Fig 1C). After glow discharge oxygen plasma treatment, a decrease in hydrophobicity was observed (Fig 1B) with a SCA of (21 ± 3)° and an increase in roughness (10 ± 3) nm, (Fig 1D), due to presence of cracks and ripping (Fig 1F). Similar results were observed by Švorčík et al. (2007), who described after argon plasma exposure an increase in 3.02 nm LDPE sheet roughness [13]. Likewise, Kikani et al. (2013) observed an increase in roughness values after submitting PE sheets to air plasma discharge, at low and high atmospheric pressures [34].

LDPE alterations (cracks and tears) are related with ablation process, where surface layers were structurally altered, due to the action of plasma ions on the LDPE, generating the formation of alkyl-, proxyl-, alkoxy- radicals [13]. High energy reactive species produce by this type of discharge (free radicals, O_3_, and other ions) hydrolyse C-H and C-C chemical bonds from LDPE surface, allowing for species containing O_2_, to react with the polymer’s carbon and form polar compounds of C-O and C = O nature [35], converting the surface from hydrophobic to hydrophylic, and decreasing CA. Same changes were described by Ataeefard et al., (2008), who detected changes in surface roughness in LDPE sheets through AFM, when exposed to Ar, O_2_, N_2_ and CO_2_ discharge [12]. Likewise, Sanchis et al. (2006), detected an increase in roughness after discharge oxygen plasma treatment [36]. The degree of LDPE modification under plasma discharge depends the voltage, pressure and discharge potency [37,38].

For FTIR a 2920 cm^-1^ signal decrease (Fig 3B) was attributed to differences in material thickness, as a response to ablation [24]. Rajandas et al. (2012) analyzed different LDPE concentrations by means of FTIR. They noted signal intensity at 2920 cm^-1^ was associated with material concentration (w/v%). Therefore, based on their observations in our work suggest pristine material exposed to O_2_ plasma discharge reduced mass, due to a higher percentage of transmitance (Table 1), [25]. Shi et al., (2006) described reactive plasma species bombarded on LDPE surface macromolecules hydrolyse C-H and C-C bonds, producing oligomers and small organic molecules. Hence, mass loss is increased [39]. Shikova et al., (2005) mention O_2_ plasma action on PE forms insaturated vinyl type groups (-CH = CH_2_), vinylen (R_1_R_2_C = CH_2_) and *trans* vinylens (-CH_2_ = CH_2_-), whose absroption bands appear between 890 and 960 cm^-1^. In this study pristine LDPE sheets presented signals in this region that were not observed in treatments. This could suggest LDPE sheets employed in this study contained different types of impurities of insaturated nature, which during pre-treatment and after fungus contact were modified. Moreover, LDPE exposed to plasma should generate carbonyl groups with signals between 1700 cm^-1^–1800 cm^-1^, however in this work they where not detected by FTIR. Last, near the 1175 cm^-1^ region a zone corresponding to C-OH group vibration stretchings was appreciated, characteristic of alcohols.

### Plackett-Burman experimental design (PB) for condition selection favoring plasma pretreated LDPE sheet biodeterioration by *P*. *ostreatus*

To favor *P*. *ostreatus* LDPE biodeterioration, a series of nutritional and operational conditions (factors) should be selected to allow fungus development. This design included glucose, copper, ABTS (redox mediator), pH and time.

In this study, SCA was a significant response variable (p = 0.049), since it had an effect on decreasing hydrophobicity influencing microorganism colonization capacity, because a hydrophilic surface is easier to colonize [4].

A slight SCA increase in PBED treatments T1 and T2 was observed. It changed from (21 ± 3) ° before plasma discharge treatment to (28 ± 6) ° (T1) and (29 ± 7) ° (T2), at 90-day incubation respectively. Contact angle gain could be due to an increment in roughness [9], the other variable in the PB with a significant response (p = 0.0011). After 90-day incubation *P*. *ostreatus* mycelia was capable of covering for the most part LDPE surface, leaving colonized and non-colonized zones that augmented CA and surface roughens (S4 Fig). It is also known polymers can recover hydrophobicity after glow discharge [9]. Previous assays (data not shown) revealed a 75.4% hydrophobicity recovery from pristine LDPE, after seven-day discharge oxygen plasma exposure under the same conditions as described in the present work. For PB hydrophobicity recovery was far less, with a 33.9% recovery for T1 and 38.8% for T2. Low hydrophobicity recovery could have been due to *P*. *ostreatus* biological activity, given hyphae network presence and fungal enzyme activity, preventing non-polar group diffusion and reorientation within the material [9]. Even though for PB design no analysis associated with fungal activity was carried-out, copper (CuSO_4_, p = 0.0136) and ABTS (p = 0.0021) were factors influencing SCA and roughness variables, respectively. These factors are related to Lac activity, since Cu^2+^ makes part of the enzyme’s active site, where substrate oxidation takes place. Moreover, ABTS mediator favors degradation of non-phenolic compounds [19], acting as an external electron carrier between laccase and the substrate to be oxidized, thus complementing enzyme activity, and reaching sites not accessible to Lac [40], oxidizing more C-C bonds, which could have favored roughness. This qualitative activity was observed in semisolid modified Radha media by green and purple halo formation, associated with oxidation metabolic processes. On the other hand, during the 90 days of PB treatment in wet chamber *P*. *ostreatus* generated pigments that diffused throughout the Radha agar resulting from oxidative stress to which the fungus was submitted (media poor in organic compounds with LDPE as a carbon and energy source).

### 150-days *P*. *ostreatus* biodeterioration assays curves. Variables associated with LDPE sheet changes (Stactic Contact angle, SCA)

LDPE sheet CA significantly changed from (87 ± 1) ° (pristine) to (21 ± 3) ° (p = 0.0139, after six-minute plasma discharge exposure. After 90-day treatment, PB T2 obtained an average SCA of (29 ± 7) °. Contact angle decrease for both cases suggests LDPE superficial tension increased, due to plasma exposure and *P*. *ostreatus* treatment. This rise is attributed to polar group formation, such as carbonyl (C = O), carboxyl (C = O-OH), and hydroxyl (-OH) [34], responsible for SCA decrease due to changes in superficial hydrophobicity [41,42]. After five-month biodeterioration curve (150 days), LDPE sheet CA for PBBT_150_ sequential treatment was 17 ± 4 ° (p < 0.0001). This could be indicating a synergic effect among plasma discharge, *P*. *ostreatus* growth and produced enzyme activity on LDPE sheets. In turn, these events could potentiate hydrophilicity on the material facilitating fungus colonization.

### Young’s modulus, yield strength and weight loss

Highest Young´s modulus values (Table 3) were obtained after PBBT_150_ and PT3 treatments, where PT3 was higher (p = 0.02252). Plasma discharge favored crosslinking and polar group incorporation to the material’s surface, hindering rotation and increasing rigidity. Likewise, *P*. *ostreatus* enzyme activity on plasma pre-treated LDPE amorphoous region generated chain hydrolysis, decreasing amorphous region proportions and increasing crystalline zones, augmenting material rigidity [43]. Similarly, in this study PT3 treatment incorporated H_2_O into Radha’s agar, favoring polar group formation, making the treated material more rigid. According to Hsu et al. (2012) the main mechanisms for degradation are carbon chain hydrolysis, principally in tertiary carbons of LDPE amorphous and amorphous/lamellar phases, where oxygen can easily diffuse into the material, while the LDPE crystalline phase remains almost unaltered [44].

The yield strength is the tension value that marks the transition from the elastic region to the plastic region and the beginning of the plastic deformation of the material. In the present study yield strength decreased with plasma treatment (PT3) and remained constant with *P*. *ostreatus* 150 day treatment (PBBT_150_), (Table 3). Chain crosslinking in the presence of polar groups on LDPE surface, as a result of plasma treatment, added to pre-treated LDPE amorphous chain biodeterioration, and carbon chains accesible to *P*. *ostreatus* enzymes maintained yield strength lower in comparison with pristine LDPE, increasing stress values delimiting the transition to the plastic region. However, *P*. *ostreatus* effect on this parameter could not be demonstrated for the 150 days of treatment.

For the 150 day biodeterioration curve a greater weight was observed for PBBT_150_ treatment (Table 3). None the less, no significant differences among treatments and controls were observed (PT3 and C2, F = 1.1397; p = 0.35385). Three scenarios could be explaining weight fluctuations. On the one hand, post-plasma ablation processes favored weight loss, as small molecular weight fragments were released [45] by action of atmospheric oxygen free radical interaction [46]. On the other hand, the material gained polarity when it oxidized, facilitating hygroscopy capturing humidity from the environment (wet chamber with semisolid agar), which could be increasing the material’s weight. Last, plasma process favored fungus colonization on LDPE, permitting greater *P*. *ostreatus* adherence to LDPE, making its release more difficult during posterior washes.

Some authors have demonstrated weight loss as an important biodeterioration variable. As a case in point, Łabużek et al., (2006) reported ˂0.04% weight loss in PE sheets after a 16 day photo-thermal degradation process (UV 250–400 nm and 50° C) [47]. Sheik et al. (2015) observed LDPE sheet weight loss (0.3 mg) after they were exposed to gamma rays greater than 200 KGy and incubaed with fungi during 90 days [21].

It is important to highlight most studies utilized tempreatures favoring water evaporation and dry environments in contrast to humid conditions in the present study. Therefore, plasma pre-treatment increased material hydrophylicity, assessed through SCA changes. This in turn increased hygroscopy, which allowed to capture water from the humid environment (wet chamber in semisolid Radha agar). Thus, weight variations were not significant, and them were not taken into account to select the best treatment. However, FTIR did detect changes in material thickness (Fig 4).

### Fourier transformed infrared spectroscopy (FTIR) analysis

According to Gulmine et al., (2002), LDPE characteristic decrease in band absorption can be attributed to differences in material thickness [24], due to ablation obtained after material was subjected to plasma discharge treatment [25]. These authors describe 2920 cm^-1^ signal intensity is proportional to LDPE concentration, which could signify material exposed to O_2_ plasma followed by fungal treament lost mass (Fig 4B).

During plasma discharge treatment, ion-electron interaction generates excited P* states that dissipate energy, breaking bonds and forming alkyl type radicals (H_3_-C·). If these radicals are in the vicinity of an LDPE polymeric chain they can generate insaturations [48], accounting for vinyl presence. Similarly, O· and ·OH radicals can remove LDPE secondary hydrogens resulting in H_3_-C· generation. LDPE C· radicals can react with O· radicals from the environment and generate peroxi- (C-O-O·) and hidroperoxi- radicals (C-O-OH), [49], as can be inferred from peaks in the region between 950 cm^-1^ and 980 cm^-1^.

SCA change implies incorporation of polar groups on the material surface, favoring LDPE moistening, thus a decrease in contant angle. Based on FITR data no band characteristic of carbonyl groups were observed (C = O region between 1700 cm^-1^ and 1800 cm^-1^), hydroxyl groups (OH, region between 3000 cm^-1^ and 3500 cm^-1^) that would favor material hydrophilicity. However, a small band on tertiary alcohol region was observed (C-OH), (Fig 3E) that could favor LDPE polarity and thus a SCA decrease.

### Carbonyl (Ico) and vinyl (Iv) indices

Polyethylene main photo-oxidation products are carbonyl and vinyl groups [7,50]. In general, many authors describe, presence of microorganisms results in increased carbonyl groups or double bond reduction [30,33,51–53]. Presence of carbonyl groups from the beginning of the experiment is noteworthy (Table 4). In theory pristine LDPE should only contain methyl groups, however, it was not the case for this material, which could be related with impurities. In T1after LDPE plasma dicharge, a slight decrease in carbonyl groups and modest increase in vinyl groups was recognized, suggesting physical treatment generated more crosslinks than oxidations on the polymeric chain.

During 150-day *P*. *ostreatus* incubation no small indices variations were observed. In BT2 during the first 30 days a minor decrease in both carbonyl and vinyl groups was detected, to later experience a similar behavior as for PBBT_150_. According to Albertsson et al., (1987), I*co* decrease occurs simultaneously as soluble carboxylic acid assimilation in LDPE biodeterioration [54]. Koutny et al. (2006) describe LDPE biodeterioration is faster in the beginning of the process, given to low molecular weight polar compound presence that are liberated into the aqueous media, after a thermos and photodegradation. In this study, plasma discharge could have generated carbonyl groups, never the less, their presence in pristine LDPE, probably associated with material quality concern, due to fabrication flaw, and a slight increase after physical treatment, could have left polar groups available for *P*. *ostreatus* availability and use [55].

### *P*. *ostreatus* growth and colonization on LDPE

LDPE sheet after plasma discharge treatment occurred on its surface the formation of carbonyl and carboxyl groups, generating on the surface a negative charge that could attract hydrogen atoms on the fungal wall [56]. Moreover, microorganisms colonizing the polymer could also have adhered to its surface by chitin and glycan production. These biological polymers covalently adhere to LDPE surface and play an important role in depolymerizing enzyme support and transport during the polymer superficial attack [33].

Similarly, it is possible to produce hydrophobins, self-aggregating proteins that form films, when exposed to hydrophobic-hydrophilic natural interphases [57]. It is also advantageous for colonization mycelial network growth and hyphae penetration [4]. As a result from colonization, the fungus generates a sequential mechanism: bioadsorption/biodeterioration [58]. Gilan, et al. 2004 specified microbial solid polymer degradation, such as LDPE, requires for the microorganism to efficiently utilize the non-soluble substrate formation of a biofilm on the polymer’s surface [10]. LDPE sheet exposed only to biological treatment presented lesser colonization. This could have resulted from high grade of hydrophobicity and a smooth surface, restricting microorganism biofilm formation, capable of polymer degradation [59].

Regarding roughness, PBBT_150_ started with high roughness value (10 ± 3) nm, as determined by AFM, and after a 150-day ablation process roughness was significantly reduced to (7 ± 2) nm ^a^ (p < 0.0025), showing the smallest variation in roughness of the biodeterioration curve, likely due to *P*. *ostreatus* colonization on the material, as was observed by SEM (Fig 4). Sheik et al., (2015), also observed *L*. *theobromae* colony formation on LDPE after gamma ray irradiation [21]. An extended growth on the sheets was observed at day 120, as evidenced by SEM images (Fig 4D). At day 150 “biofilms” were released, leaving areas with and without growth, creating great roughness (Fig 4E).

Treatment PT3 presented the greatest variation in roughness, changing from 10 ± 1 nm to 4 ± 1 nm at day 150. For this treatment LDPE only underwent plasma discharge treatment. As has been previously described, it could have presented hydrophobic recovery, since post-plasma modification was not permanent [9]. Migration of low molecular weight species from the “bulk” to the surface, in addition to surface roughness relaxation, are responsible for hydrophobic recovery [60]. Furthermore, plasma exposure time (six minutes), could have also had an effect on recovery. Hegemann et al., (2003), reported short plasma discharge generate a break on external chains, while longer exposures can break internal polymer chains, increasing roughness and the time of material recovery [61].

Regarding variables associated with fungal metabolism, for PBBT_150_ Pearson correlation were positive for biomass and LiP activity (ρ = 0.97, p < 0.0001) and pigment production (ρ = 0.83, p = 0.0446). It could be inferred they are closely associated and greater biomass production induced higher LiP activity and increased pigment formation, as was evidenced from day 30 to day 150. In bioremediation processes performed with WRF, contaminant structure changes are generally associated with extracellular enzyme and biomass production [62]. Dark brown pigment synthesis reflects a plausible fungal defense mechanism, against oxidative stress, produced during the ablation pre-treatment and free radical production by the fungus.

Lac and MnP activities did not have a positive correlation with biomass, which could be related to redox mediator consumption, since both enzymes initiated with high values, yet after 30-day a decrease in activity was observed, in comparison to other sampling times. These data suggest redox mediator was rapidly depleted, thus decreasing enzyme oxidation potential. However a periodic addition could favor an increase in activity and its action on LDPE.

When relating metabolism associated variables with some LDPE physical and chemical property changes, some significant positive correlations were obtained. Examples include between biomass and carbonyl index (ρ = 0.580, p = 0.0480), biomass and vinyl index (ρ = 0.6461, p = 0.0430). Moreover, between LiP activity and carbonyl index (ρ = 0.5425, p = 0.0042), and between Lac activity and carbonyl index (ρ = 0.540, p = 0.0493). Even though enzyme activities were not high, complementary oxidation processes by plasma discharge were undergoing, since they were producing a greater quantity of carbonyl groups that could be susceptible to successive oxidations until production of carbonyl groups was reached. Most likely a time surpassing 150-day is required, since the present study utilized non oxobiodegradable commercial plastic, making it more resistant to oxidation.

The only enzymes presenting a significant negative correlation with decreased CA were MnP (ρ = -0.4826, p = 0.0169) and LiP (ρ = -0.4876, p = 0.0155). This indicates an increasing activity favors CA decrease, hence maintaining LDPE hydrophilicity. This process in term promotes fungus colonization and oxidation. For surface roughness only a significant positive correlation was evidenced with MnP activity (ρ = 0.914, p = <0.0001).

Biological treatment analysis of BT2, evidenced correlations were found between biomass production and Lac activity (ρ = 0.7048, p = 0.0105), biomass production and MnP activity (ρ = 0.8437, p = 0.0006), and biomass production and LiP activity (ρ = 0.9369, p < 0.0001). Furthermore, a significant correlation was observed between biomass and pigment production (ρ = 0.7340, p = 0.0066). For certain time intervals these data reveal biomass quantity determines extracellular enzyme activities, independent from previous LDPE plasma discharge treatments. Additionally, Lac and MnP activities presented positive significant correlation (ρ = 0.7121, p = 0.0094). Even though they were the least volumetric activity, they have a tendency to increase in a similar manner, particularly from day 60.

Similar results were obtained for BT2 I*co* as for PBBT_150_. Positive significant correlations were observed between I*co* and biomass (ρ = 0.6933, p = 0.0124), I*co* and Lac activity (ρ = 0.6010, p = 0.0387), I*co* and MnP activity (ρ = 0.8577, p < 0.0001, I*co* and LiP activity (ρ = 0.7002, p = <0.0001). These results prove *P*. *ostreatus* can undergo LDPE adsorption, adhesion, colonization, and oxidation processes to produce carbonyl groups without needing plasma discharge pre-treatment. However, LDPE could recover some of its hydrophobic properties, since CA at 150 days was 49 °, i.e. 2.92 times higher in comparison with plasma pretreatment. For LDPE biodeterioration previously described results experimentally support that described by other authors, where various enzymatic mechanisms take place. Never the less, for *in vitro* conditions it is necessary to favor biomass colonization and lignolytic enzyme production.

Contact angle was negatively associated with biomass. However, MnP and LiP activities were not significant (p > 0.05).

### *P*. *ostreatus* associated response variables

*P*. *ostreatus* growth in PBBT_150_ was 1.94 greater compared with BT2 (Fig 4), demonstrating discharge plasma treatment facilitated LDPE colonization. For *P*. *ostreatus* to be able to carry out biodeterioration processes first it must adsorb its hyphae to the material surface, followed by their adhesion by exopolysaccharide production and material colonization, which later allows for LDPE degradation by extracellular enzymes of peroxidase- (E.C. 1.11.1.7) and oxidase polyphenol type (E.C. 1.14.18.1) that hydrolyze C-C bonds, with participation of redox mediators.

Semi-quantitative ABTS oxidation was evidenced by green halo formation (first oxidation state) and purple halo formation (second oxidation state) in the same time periods for PBBT_150_ and BT2. These results suggest from the beginning Lac and ABTS could be acting on high molecular weight material, such as plastic. In addition, ABTS should be added with certain periodicity to favor Lac activity, because as it will be evidenced later, its greatest activity was detected during the first 30 days.

White rot fungi during lignin degradation can produce oxidative intermediates that can be toxic to them; however they inactivate them by polymerization in melanin-type pigments [63]. According to Toledo et al., (2017), melanins could play the role as an extracellular redox buffer to neutralize oxidative agents generated by environmental pressures [64]. The presence of pigments in this study could be associated with free radicals protection mechanism that could be formed particularly during ablation process, since it is an ionization discharge. Furthermore, some lignolytic fungi, such as *P*. *ostreatus*, *T*. *versicolor* and *P*. *chrysosporium* also increase these type of pigment production in presence of dyes or heavy metals and polyurethane [65]. Madhavi & Lele, (2009) describe yellow laccases are capable of oxidizing non-phenolic lignins [66]. It was observed in PBBT_150_ (S2 Fig) yellow zones around seeded biomass, different from BT2 that presented brown zones. It is possble that yellow laccases were also produced in this sutdy.

In PBBT_150_ *P*. *ostreatus* LiP activity was high between day 90 and 120, it descended at day 150. In contrast, BT2 activity was constant during the same time period. Last, at day 150 thick mycelia was evidenced as a product of stress for PBBT_150_ and BT2, as well as for C3. Lee et al., (1991) described veratryl alcohol test to determine *Phanerochaete* sp. LiP activity in 3% malt extract (w/v) liquid culture revealed loss of color in culture media associated with ligninase activity (E.C.1.11.1.14). On the other hand, cultures that retained their color did not produce such enzyme [67]. In the present study, although the media was semisolid the same phenomenon could have presented during biodeterioration curve, where loss of color was observed for BT2 biomass after day 60.

As described by Majeau et al., (2010) Lac production increases, when glucose becomes a limitant [68]. In this study from the beginning *P*. *ostreatus* growth in wet chamber was favored by the presence of simple carbon and nitrogen sources (0.625gL^-1^ glucose and 0.05gL^-1^ NH_4_Cl), at low concentrations that benefit growth, colonization and enzyme production, in particular Lac during the first 30 days [69], when they act as co-substrates to support primary metabolism, while physical, chemical and biological porcesses take place to biodegrade LDPE sheets. For biodeterioration of these type of plastic, whereas in liquid or solid culture, in soil or with lignocellulosic materials, more simple organic compounds must be added to allow initial fugus growth before it can use the carbon coming from the plastic [6].

Regarding enzyme activity, Lac participated in LDPE biodeterioration, as it oxidizes the main LDPE C-C chain with aid of ABTS type mediators [70]. High *P*. *ostreatus* Lac activity at day 30 could have been related with presence and initial ABTS redox mediator oxidation, present in Radha semisolid media. *P*. *ostreatus* Lac redox potential is 0.65 V, relative to hydrogen standard potential, which is low, thus it cannot completely oxidize C-C bonds, which has a higher redox potential, whereas ABTS has a 0.8 V redox potential, which favors C-C bond hydrolysis, increasing the oxidative capacity of the enzyme [71]. This activity has also been determined in solid matrices, where *P*. *ostreatus* produced 185.5 Ug^-1^ in mineral paper and LDPE oxobiodegradable in a period of 45 days [17].

In the present study biomass was washed and centrifuged in glucose solution and ammonium chloride (co-substrates of carbon and nitrogen, respectively), which might explain why laccase activity was only observed during the first 30 days of incubation. Moreover, it could also explain Lac activity during this time period, due to ABTS mediator depletion, since it was only added at the beginning, and could have limited enzyme activity after its depletion. Additionally, semisolid media contained 1.5 mM CuSO_4_ favoring *P*. *ostreatus* laccase activity [72].

Lac activity has been reported for diverse fungi exposed to LDPE. As a case in point, *Lasidioplodia theobromae*, reported a 10.70 UmL^-1^ laccase activity in LDPE media submitted to UV radiation [73]. Likewise, some marine fungi present activity on pristine LDPE, these include: *Eupenicillium hirayamae* 17.00 Um L^-1^, *Paecilomyces variotii* 12.68 UmL^-1^, *Alternaria alternata* 2.60 UmL^-1^ and *Phialophora alba* 8.69 U mL^-1^ [15]. In this study the highest Lac activity was observed at day 30 for treatment PBBT_150_, with an activity of 2.187 ± 0.346 U Lac g^-1^.

In regards to MnP and LiP enzyme, no reports describe their mechanisms in LDPE biodeterioration [18]. Therefore, we assume this process might occur in a similar manner as for lignin biotransformation, where these enzymes employ H_2_O_2_ to catalyze phenolic fraction oxidation, non-phenolic and aromatic rings respectively [74]. Jiménez et al. (1999) report MnP and LiP enzymes are regulated by the presence of carbon and nitrogen in the culture media [75]. Janusz et al. (2013) describe high *P*. *ostreatus* Lac and MnP enzyme production can be influenced by high nitrogen concentrations [76]; which could have favored Lac and MnP activities at day 30 for PBBT_150_, presenting the highest values for the experiment (2.8167 ULac g^-1^ and 0.862 Ug^-1^), respectively. Additionally, MnP possesses a redox potential about 0.8 V, which allows to oxidize C-C bonds without mediator participation [55].

When evaluating MnP activity it was predominant at 30 and 150 days for PBBT_150_ (0.862 Ug^-1^ and 0.236 Ug^-1^, respectively) and for 60, 90, and 120 days it was predominant at BT2 (0.665 Ug^-1^, 0.543 Ug^-1^ and 1.097 Ug^-1^). Iiyoshi et al. 1998 evidenced for PE biodeterioration under limited carbon and nitrogen conditions in *Phaneochaete chrysosporium* (18.0 Ug^-1^) and *Trametes versicolor* (4.9 Ug^-1^) fungi MnP activity. They point out given enzyme’s Mn^2+^ requirements; its concentration in the media might affect MnP production [74].

Jiménez et al., (1999), described Mn^+2^ presence is necessary for MnP activity, yet its excess inhibits LiP. In the present study Radha semisolid modified media contained trace microelements (10 mL L^-1^), among them manganese chloride (II), (0.1 gL^-1^ MnSO_4_), which could have favored MnP production, yet not inhibiting LiP. This later enzyme presented the highest activities in this study at days 90 and 120 with 70.755 Ug^-1^ and 68.820 Ug^-1^ respectively for PBBT_150_. For BT2 LiP activity presented a constant increase without surpassing 40 Ug^-1^ at day 150. This suggests, sequential process, i.e. plasma + fungus (PBBT_150_) favored LiP expression. LiP has an ample redox potential (1.2 V relative to standard hydrogen potential), which allows it to oxidize non-phenolic compounds, even without the presence of a mediator [77]. This aspect could have favored LDPE oxidation after 30 days, particularly between days 90 and 120, when Lac activity was already decreased due to ABTS depletion. In the same manner, LiP activity could have permitted *P*. *ostreatus* colonization.

The only report regarding activity of this enzyme acting on LDPE is from Ameen et al., (2015). They reported *A*. *alternata* (6.15 UmL^-1^), *Aspergillus caespitosus* (9.98 UmL^-1^) and *P*. *alba* (2.4 UmL^-1^) activity obtained from swamp [78].

Lingnolytic fungi, such as *P*. *ostreatus* not only produce enzymes to degrade lignin, they also generate a series of free radicals, such as peroxides, superoxides and hydroxyls, which act as oxidation diffusible mediators for PE degradation [55].

Li et al, (1999) proposed when 1-hydroxybentrotriazole (HBT) mediator was oxidized by Lac, released radicals can oxidize non phenolic compound in lignin [79], suggesting formed radicals from plasma discharge as well as from *P*. *ostreatus* enzyme activity facilitated LDPE degradation. Last, Table 6 illustrates LDPE percentage changes after PBBT_150_ treatment.

**Table 6:** Variable data associated with LDPE sheets revealing the greatest changes after PBBT_150_ treatment.

Variable	Pristine (100%)	*P*. *ostreatus* 150-day treatment	% Change after *P*. *ostreatus 150 days treatment*
Static contact Angle (°)	87 ± 1	17 ± 4	81
Young modulus (Mpa)	34 ± 6	48 ± 4	28
Yield strength (MPa)	10 ± 2	8 ±1	26
Roughness (nm)	5 ± 1	7 ± 2	33

Once PBBT_150_ was completed Table 6 demonstrates SCA and yield strength decreased, while roughness and Young´s modulus increased in comparison with prostine, favoring material deterioration.

## Conclusions

Sequential treatment (glow O_2_ discharge plasma followed by *P*. *ostreatus* adherence, growth, and colonization) after 150 days of the biodeterioration curve modified surface physical characteristics, increasing hydrophilicity and augmenting surface roughness, at the same time that *P*. *ostreatus* showed a high lignolytic activities, with concomitant high pigment release of melanin type, supporting metabolic stress when exposed to the polymer. The ~ 27% of changes in mechanical properties of the LDPE (Young´s modulus and yield strength) after sequential treatment proposed in this paper support too clearly a material biodeterioration.

## Materials and methods

### LDPE sheets characteristics and cleaning

LDPE was obtained in Bogotá, D.C., Colombia local commerce. Rectangular sheets of 3.0 ± 0.1 cm x 1.0 ± 0.1 cm were cut, with an initial weight of 5.0 ± 0.7 mg and 16 ± 2 μm thick (ASTM D5947-11). Calculated plastic density was (0.87 ± 0.02) g m^-3^. Before use, sheets were washed with 99.8% methanol (v / v) for 2 minutes and rinsed with distilled water and dried for 15 minutes at 14° C [73].

### Pretreatment of LDPE with oxygen glow discharge plasma

A low temperature plasma (LTP) was generated in a cylindrical jug-type chamber, 18 cm high by 18 cm in diameter with disk electrodes separated 5.6 cm from each other and coupled to a vacuum turbo molecular pump (Pfeiffer Vacuum). Clean LDPE sheets were placed at the anode and were physico-chemically treated with oxygen low discharge plasma (O_2_, 3.0 x10^-2^ mbar, 600 V) for six minutes; previously standardized conditions (data not shown), based on two considerations: (i) that the plasma would not burn or entwine the LDPE sheets and (ii) voltage conditions as a function of the pressure would generate a stable plasma.

### *Pleurotus ostreatus* reactivation and culture media propagation

*P*. *ostreatus* was obtained from microorganism collection at the “*Laboratorio de Microbiología Ambiental y de Suelos*, *Pontificia Universidad Javeriana*, *Bogotá*, *D*.*C*., *Colombia*”. Reactivation was carried-out in wheat-bran extract agar in (175 gL^-1^ wheat-bran, 10 gL^-1^ glucose, 2 gL^-1^ yeast extract, 5 gL^-1^ peptone, 0.05 gL^-1^ MgSO_4_·7H_2_O, 0.076 gL^-1^MnSO_4_·H_2_O, 0.1 gL^-1^KH_2_PO_4_, 0.1 gL^-1^ chloramphenicol, 20 gL^-1^ agar-agar) seeded on an agar disc grown with the fungus, in the center of a Petri dish incubated for seven days at 28 °C. For production of pelletizing biomass a 250 mL Erlenmeyer flask containing 130 mL wheat-bran broth supplemented with 0.1 gL^-1^ chloramphenicol was inoculated with ten agar discs with *P*. *ostreatus* fungal biomass. Erlenmeyer flasks were incubated for ten days at 120 rpm and 25 °C in orbital shaker (New Brunswick Scientific ^™^ Innova 44). After incubation culture was centrifuged at 9790 x *g*, for 10 minutes, at 4 °C in Sorvall^™^ RC 6 plus. Biomass was washed 5 times with 0.625 gL^-1^ glucose and 0.050 gL^-1^ ammonium chloride solution.

### Plackett-Burman experimental design (PB) for condition selection favoring plasma pretreated LDPE sheet biodeterioration by *P*. *ostreatus*

To select conditions favoring *P*. *ostreatus pretreated* LDPE colonization and/or biodeterioration a Plackett-Burman (PB) experimental design was performed with five factors at two levels (+1; -1) and three central points (Table 7). PB experimental design was carried out in a wet chamber system. As control non-pretreated LDPE sheets were used. Results were rated with an empiric model to relate quantified responses with evaluated factors and their respective levels. For PB the first order model was (Eq 1):

$$Y=\beta_{0}+\sum \beta_{i}X_{i}$$ (1)

**Where**: *Y* is the result, *βo* is the model intercept and *βi* is the estimated coefficient for each *Xi* factor.

**Table 7:** PB experimental design for *P*. *ostreatus* treatment of plasma pretreated LDPE.

Treataments	Glucose (gL^-1^)	CuSO_4_ (mM)	ABTS (mM)	pH	Incubation time (Days)
**T1**	1.0	2.0	0.2	4.5	90
**T2**	0.25	2.0	0.1	7.0	90
**T3**	1.0	1.0	0.2	7.0	90
**T4**	0.25	2.0	0.2	7.0	30
**T5**	0.25	1.0	0.1	4.5	90
**T6**	0.25	1.0	0.2	7.0	90
**T7**	1.0	1.0	0.1	7.0	30
**T8**	1.0	2.0	0.1	4.5	90
**T9**	1.0	2.0	0.1	7.0	30
**T10**	0.25	2.0	0.2	4.5	30
**T11**	1.0	1.0	0.2	4.5	30
**T12**	0.25	1.0	0.2	4.5	30
**Central Points**	0.625	1.5	0.15	5.75	60
	0.625	1.5	0.15	5.75	60
	0.625	1.5	0.15	5.75	60

### Wet chamber system employed for *P*. *ostreatus* PB experimental design for pretreated LDPE biodeterioration

To implement PB and biodeterioration curves a wet chamber system was designed employing modified semisolid Radha agar [80], (2 gL^-1^ KH_2_PO_4_, 0.05 gL^-1^NH_4_Cl, 0.5 gL^-1^ MgSO_4_·H_2_O, 0.1 gL^-1^CaCl_2_·2H_2_O, trace element solution (0.5 gL^-1^MnSO_4_, 0.1 gL^-1^FeSO4·7H2O, 0.1 gL^-1^ZnSO_4_·7H_2_O), 10 mL L^-1^, 7 gL^-1^agar-agar); glucose, CuSO_4_ and redox mediator (ABTS), were employed at different concentrations according to combinations generated by PB (Table 7). This media was used as a support for LDPE sheets, using sterile Petri dishes (90 mm diameter), where 20 mL Radha agar was poured into at 45 °C and allowed to jellify for 20 minutes. Once solid, 3 cm^2^ rectangles were cut out and LDPE sheets were deposited (plasma treated and untreated) with 1 g wet viable fungus biomass (S5 Fig). Treatments were incubated at 28 °C in VELP^™^ Scientific FOC 225I incubator. After incubation period, according to PB treatments, sheets were removed, submerged in water at 20 °C with slight agitation, to release adhered *P*. *ostreatus* biomass. To evaluate PB treatment effect on the material, physical and chemical changes were analyzed (see *response variables associated with LDPE sheet changes*).

### 150-days *P*. *ostreatus* biodeterioration assays curves

Best PB experimental design treatment (renamed as Placket Burman best treatment; PBBT_150_) was used for 150-days *P*. *ostreatus* biodeterioration assays curves. Using the same methodology as in PB and culture media, a new wet chamber set-out was established to perform biodeterioration curves. Treatments and controls were: PBBT_150_: Physical/biological treatment (LDPE + plasma + *P*. *ostreatus*); BT2: biological treatment (LDPE + *P*. *ostreatus*); PT3: physical treatment (LDPE + plasma); C1: LDPE in wet chamber; C2: pristine LDPE and C3: *P*. *ostreatus* in modified semisolid Radha agar. Assays were performed in triplicates and samples were collected by sacrifice of the experimental units at 0, 30, 60, 90, 120 and 150 days. During the assay response variables associated with fungus metabolism were determined (see response variables associated with *P*. *ostreatus* metabolism) and to LDPE physical and chemical changes (see response variables associated with LDPE sheet changes).

### Variables and analytical techniques associated with LDPE sheet changes

The following variables (weight, Young’s modulus, yield strength, contact angle, topology, roughness, chemical structure, carbonyl number and vinyl index) were measured as pristine LDPE (C2), LDPE after plasma treatment (PT3) and plasma pre-treated LDPE after *P*. *ostreatus* treatment (PBBT_150_). As response variables in PB only weight, Young’s modulus, yield strength, roughness and contact angle were included.

### Weight loss

Weight loss rate was determined by direct measurement with an Ohaus Explorer analytical balance (0.1 mg resolution).

### Viscoelastic properties

LDPE sheet yield strength [81] and Young’s modulus [82] were determined from stress-strain curves [83], (Maxwell model on approximation of small deformations) with a "speed of testing" of 0.1 mms^-1^, maximum strain of 250%, and maximum force of 5 N.

### Hydrophobicity

Surface treatments with plasma have a strong effect on surface tension increase and on LDPE chemistry and surface topology [84]. Additionally, it has been shown that LDPE biodegradation depends to a large extent on hydrophilicity, thus static contact angle (SCA) was determined, a fundamental parameter that determines both hydrophilicity and surface tension [85].

To define LDPE hydrophobicity changes static contact angle (SCA) variation at room temperature (RT 14° C) was measured [80,84], between a drop (50 μL) of distilled water and LDPE surface by means of sessile drop method and recording with a videocamara (JVC^™^ GZ-EX355 Everio). To this end 50 μL deonized water was placed on top of the sheets and photographs were captured at three points of the sample [34]. Contact angle was calculated according to Eqs 2 and 3 (mean ± SD) [86].

$$\propto ={\text{sin}}^{-1}(\frac{a}{R})$$ (2)

$$R=(a^{2}+h^{2})/2h$$ (3)

**Where**: *R* is the segment of the radius of the sphere that describes the drop of water; *a* drop of water radius and *h* drop of water height.

### Fourier transformed infrared spectroscopy (FTIR) analysis

Fourier transformation infrared spectroscopy was used to evaluate LDPE chemical bond modifications and chemical group composition [34,73]. AShimadzu IR Prestige-21 spectrophotometer was employed with the following parameters: Measurement Mode: % transmittance, Apodization: Happ-Genzel, No. of scans: 20, Resolution: 4.0cm^-1^, Range: 400–4,000cm^-1^.

Carbonyl index (I*co*) was calculated by a ratio between 1718cm^-1^ absorption band (A_1718_) and 1377 cm^-1^ band (A_1377_), (Eq 4). A_1718_ corresponded to carbonyl group extension vibration (C = O), and A_1377_ was the reference, since it did not present changes during treatments [87]. Likewise, vinyl index (I*v*) was calculated as the ratio between 909 cm^-1^ (A_909_) absorption band and 2020 cm^-1^(Eq 5). Vinyl group extension vibration (CH_2_ = CH)n was A_909_ and the reference 2020 cm^-1^ absorbance, since it did not present any changes during treatments [51].

$$Ico=\frac{A_{1718}}{A_{1377}}$$ (4)

$$Iv=\frac{A_{909}}{A_{2020}}$$ (5)

### Surface morphology analysis

*P*. *ostreatus* colonization and LDPE sheet roughness was evaluated through scanning electron microscopy (SEM, Jeol JSM 6490LV), [35, 73] and atomic force (AFM, Nano surf easy scan 2) respectively. In AFM the Arithmetic Average Superficial Roughness [49, 84, 88] of the area analyzed was determined and the measurements obtained were averaged in three different places of the LDPE sheet.

Last, atomic force microscopy (AFM), [49,84] and scanning electrom microscopy (SEM), [35,73], were used to study surface changes (roughness and topography). Nanosurf ^™^ easyscan 2 contact mode was employed for AFM. Parameter were: size: 61.8 μm, Set point: 20 nN; P-Gain: 1000; I-Gain: 100; D-Gain: 0. For roughness calculation, three measurements at different locations of the sample were carried-out, and mean ± SD was determined according to Eq 6 [88].

$$R=\frac{1}{N}\sum_{i=1}^{N}\left|Zi-\overset{-}{Z}\right|$$ (6)

**Where**: *N* is surface height data number and $\overset{-}{Z}$ mean height distance.

Additionally, SEM (Jeol^™^ JSM 6490LV) with a 10 kV to 20kV potency SEI signal and 500 and 6,500 X was used to characterize surfaces.Servicecontractedthrough Universidad de los Andes (UNIANDES), Bogotá, Colombia. Samples were coated with gold in Denton Vacuum Desk IV preparation system.

### Response variables and analytical techniques associated with *P*. *ostreatus* metabolism

The following response variables (biomass, laccase activity (Lac, EC. 1.10.3.2), manganese peroxidase activity (MnP, EC. 1.11.1.13), lignin peroxidase activity (LiP, EC 1.11.1.14) and diffusible pigments) were measured to the plasma pre-treated LDPE after 150 days of *P*. *ostreatus* treatment (PBBT_150_); as control *P*. *ostreatus* growing in Radha media was used (C3).

### Biomass and diffusible pigment

Biomass production as a function of time [65], appearance of diffusible pigment in the media, and laccase semiquantitative activity were evaluated weekly for 150 days. Distances were determined in mm, for the mycelium, enzyme activity halos, and diffusible pigments from the edge of the Petri dish to the border of the biomass (S6 Fig).

### Ligninolitic enzymes activities

For laccase (Lac), manganese peroxidase (MnP) and lignin peroxidase (LiP) enzyme activities within the wet chamber it was necessary to extract enzymes using distilled water (23 mL/Petri dish) and liquefy semisolid agar in Hamilton Beach^™^ food processor for two minutes to homogenize the sample [28]. The product was filtered through Whatman No. 3 filter paper and used as extract to evaluate enzyme activities in triplicates.

Lac activity was determined at pH 5.0 in 600 mM acetate buffer, by monitoring ABTS oxidation (2,2'-azino-bis(3-ethylbenzothiazoline-6-sulphonic acid)) at λ_436 nm_ for three minutes at room temperature (RT, 14 °C). Reaction volume was 1,000 μL, containing 800 μL extract, 100 μL 600 mM acetate buffer (1.306% w/v acetic acid and 5.226% w/v sodium acetate) pH 5.0 and 100 μL de 5 mM ABTS (Sigma-Aldrich). A laccase activity unit (U Lac) is the quantity of enzyme required to oxidize 1 μmoLABTS in 1 minute [89].

MnP activity was quantified by 2,6 dimethoxyphenol (DMP)oxidation in 100 mM acetate buffer pH 5.0by change in absorbance at λ_468 nm_ during three minutes at room temperature (RT, 14 °C). Quantification was carried-out by mixing 450 μL extract, 500 μL 10 mM DMP (Sigma-Aldrich), 50 μL 0.4 mM MnSO_4_ and 30 μL 22 mM H_2_O_2_. A MnP activity unit (UMnP) is the quantity of enzyme required to oxidize 1 μmoL DMP per minute [90].

LiP activity was quantified by oxidation of veratryl alcohol in 10 mM sodium tartrate buffer [(0.803% (m/v) tartaric acid and 4.464% (m/v) sodium tartrate]. Activity was determined by monitoring change in absorbance at λ_310 nm_ for three minutes at room temperature (RT, 14 °C). Reaction mix contained 710 μL extract, 200 μL tartrate buffer, 50 μL 8.12% (v/v) H_2_O_2_ and 40 μL 3.65% (v/v) veratryl alcohol (Sigma-Aldrich). A LiP activity unit (U LiP) is the quantity of enzyme required to oxidize 1 μmoL veratryl alcohol per minute [84].

Semi-quantitative and quantitative analyses were determined by Shapiro-Wilk normality test and Levenne’s variance homogeneity with SPSS^®^ V22.0 for Macintosh. To evaluate differences among curves analysis of variance was performed (ANOVA). A Tukey *post hoc* test was carried-out to establish differences among means.

### Computational image processing

Computational image processing techniques were used to measure *P*. *ostreatus* colonization on LDPE surface from obtained SEM images. All images were formatted to 1280 x 860 pixels and their noise was reduced by a Gaussian filter aided by an Euclidean curve shortening flow process to enhance edges [91]. The brightest sections were selected using the morphological Top-Hat transformation [92]. Lastly, a binary process based on morphology was used to select pixels, whose intensity was above a base threshold. The colonized area was calculated as the number of pixels selected divided by the total number of pixels in the image.

### Statistical analysis

For ANOVAdata analysis Desing Expert V. 9.0 software was employed for Plackett-Burman design. Comparison among treatment means was carried out using Tukey *post hoc* test (p < 0.05) SAS 9.0^®^. Additionally, to determine relevant associations among evaluated variables a correlation matrix was constructed and Pearson correlation was performed taking into account 150 days of the process. Data were analyzed using SAS 9.0 with a 5% (α = 0.05) significance level for all tests.

## Supporting information

S1 Fig Plackett Burman experimental design. Petri dishes containing semisolid modified Radha media, *P*. *ostreatus* biomass, and LDPE sheets at (**A**) day 0, (**B**) day 60, and (**C**) day 90.
(DOCX)

S2 Fig 150-day biodegradation curve. *P*. *ostreatus* on semisolid modified Radha media and LDPE sheets **C3**: *P*. *ostreatus* in semisolid modified Radha media (control). **PBBT**_**150**_: Physical treatment followed by biological treatment (plasma discharge pre-treated LDPE followed by fungal biodegradation). B**T2**: Biological treatment (LDPE only with fungus treatment). Metabolic stress can be observed after five-month incubation in contact with LDPE. Thick mycelium, diffusible pigment release, and loss of biomass color.
(DOCX)

S3 Fig LDPE I*co* and I*v* índices during 150-day biodegradation curve. PBBT_150_: Physical/biological treatment. BT2: Biological treatment.
(DOCX)

S4 Fig Atomic force microscopy (AFM). LDPE with *P*. *ostreatus* growth at day 90. Hyphae on material surface.
(DOCX)

S5 Fig Wet chamber with Radha semisolid modified media. (**A**) *Pleurotus ostreatus* growth in wheat bran broth. (**B and C**) LDPE sheets and *P*. *ostreatus* biomass set-up of in Radha semisolid modified media. (**D**) Laccase enzyme production set-up by oxidation (green halos) of ABTS redox mediator. (**E**) *P*. *ostretus* after 60 days of growth with Radha semisolid modified media in contact with LDPE sheets.
(DOCX)

S6 Fig Growth measurement, pigment production and semi-quantitative laccase production in Radha semisolid modified media after 150-day treatment (biodegradation curve performed in wet chamber). *P*. *ostreatus* distance (in mm) was measured weakly from the exterior edge of the biomass (longest side) to agar with brown halo, mycelium, pigment (light brown areas) and enzyme activity (green or purple zones). Mycelium was measured by examining the bottom of the Petri dish. The same methodology was used for pigment and semiquantitative enzyme activity.
(DOCX)
