# Supplementary figures and images for: Biodeterioration of plasma pretreated LDPE sheets by Pleurotus ostreatus
Source: PLoS One. 2018 Sep 13;13(9):e0203786. doi: 10.1371/journal.pone.0203786 (PMC6136779; doi:10.1371/journal.pone.0203786)

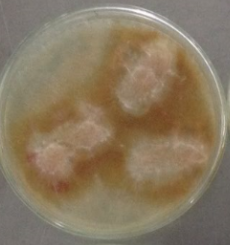

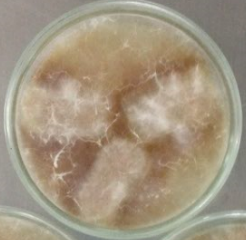


**A**

**B**

**C**

Supplement: S1 Fig — Petri dishes containing semisolid modified Radha media, P. ostreatus biomass, and LDPE sheets at (A) day 0, (B) day 60, and (C) day 90. (DOCX) [file pone.0203786.s001.docx]

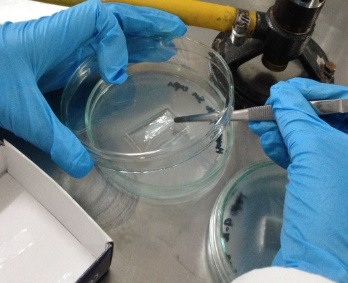

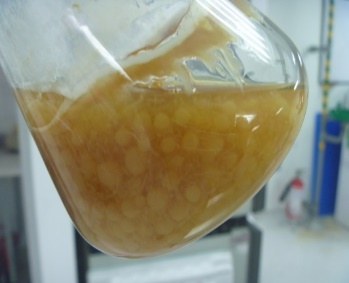

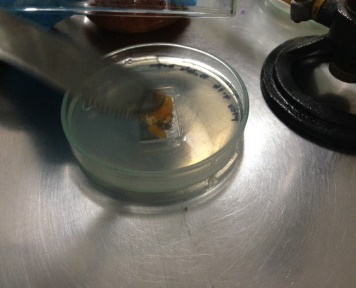

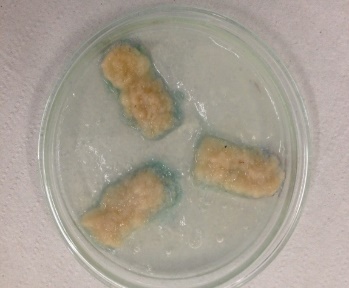


**A**

**C**


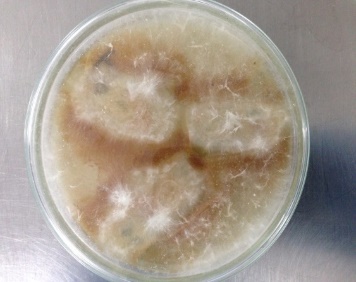


**E**

**B**

**D**

Supplement: S5 Fig — (A) Pleurotus ostreatus growth in wheat bran broth. (B and C) LDPE sheets and P. ostreatus biomass set-up of in Radha semisolid modified media. (D) Laccase enzyme production set-up by oxidation (green halos) of ABTS redox mediator. (E) P. ostretus after 60 days of growth with Radha semisolid modified media in contact with LDPE sheets. (DOCX) [file pone.0203786.s005.docx]

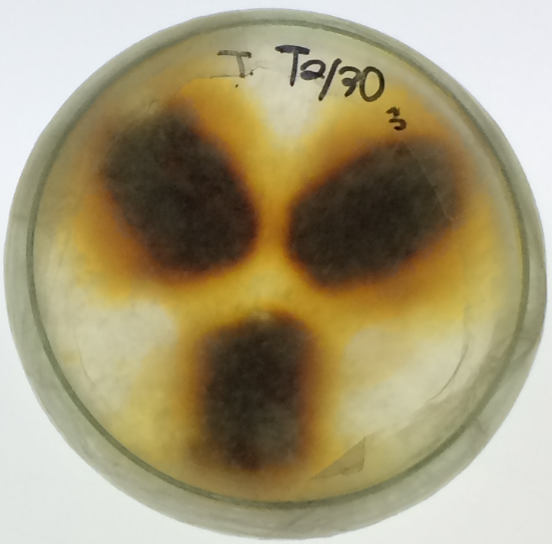


mm

mm

Supplement: S6 Fig — P. ostreatus distance (in mm) was measured weakly from the exterior edge of the biomass (longest side) to agar with brown halo, mycelium, pigment (light brown areas) and enzyme activity (green or purple zones). Mycelium was measured by examining the bottom of the Petri dish. The same methodology was used for pigment and semiquantitative enzyme activity. (DOCX) [file pone.0203786.s006.docx]
