# Supplementary material for: Biodeterioration of plasma pretreated LDPE sheets by Pleurotus ostreatus
Source: PLoS One. 2018 Sep 13;13(9):e0203786. doi: 10.1371/journal.pone.0203786 (PMC6136779; doi:10.1371/journal.pone.0203786)
Supplement: Supplementary file 2 [file pone.0203786.s002.docx]

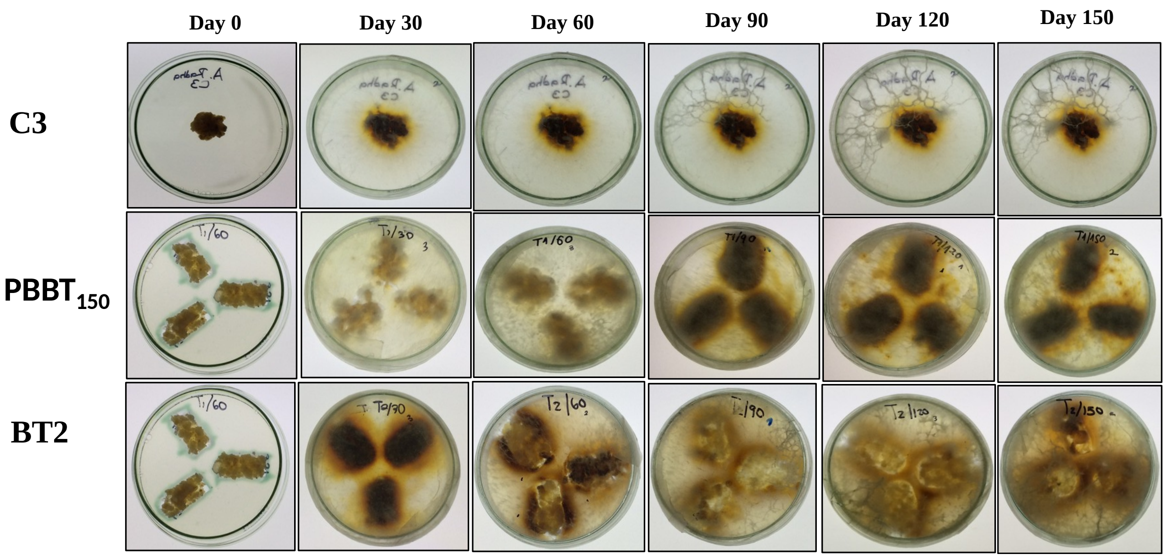

Day 0
Day 30
Day 60
Day 90
Day 120
Day 150
C3
PBBT150
BT2
