# Supplementary material for: Biodeterioration of plasma pretreated LDPE sheets by Pleurotus ostreatus
Source: PLoS One. 2018 Sep 13;13(9):e0203786. doi: 10.1371/journal.pone.0203786 (PMC6136779; doi:10.1371/journal.pone.0203786)
Supplement: Supplementary file 4 [file pone.0203786.s004.docx]

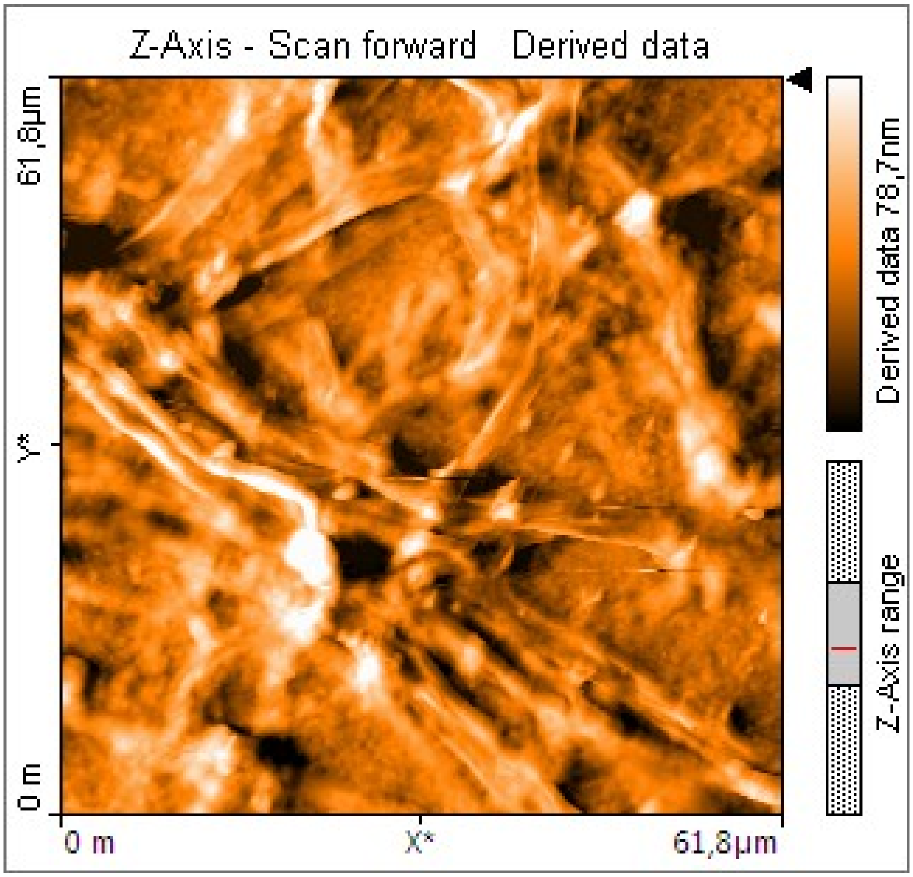
Z-Axis - Scan forward Derived data
61,8µm
Y*
0 m
0 m
X*
61,8µm
Derived data 78,7nm
Z-Axis range
